# Integrative systems biology reveals NKG2A-biased immune responses correlate with protection in infectious disease, autoimmune disease, and cancer

**DOI:** 10.1016/j.celrep.2024.113872

**Published:** 2024-02-29

**Authors:** Daniel G. Chen, Jingyi Xie, Jongchan Choi, Rachel H. Ng, Rongyu Zhang, Sarah Li, Rick Edmark, Hong Zheng, Ben Solomon, Katie M. Campbell, Egmidio Medina, Antoni Ribas, Purvesh Khatri, Lewis L. Lanier, Philip J. Mease, Jason D. Goldman, Yapeng Su, James R. Heath

**Affiliations:** 1 Institute of Systems Biology, Seattle, WA, USA; 2 Vaccine and Infectious Disease Division, Fred Hutchinson Cancer Research Center, Seattle, WA, USA; 3 Clinical Research Division, Program in Immunology, Fred Hutchinson Cancer Research Center, Seattle, WA, USA; 4 Molecular Engineering & Sciences Institute, University of Washington, Seattle, WA, USA; 5 Department of Bioengineering, University of Washington, Seattle, WA, USA; 6 Institute for Immunity, Transplantation and Infection, School of Medicine, Stanford University, Stanford, CA, USA; 7 Center for Biomedical Informatics Research, Department of Medicine, School of Medicine, Stanford University, Stanford, CA, USA; 8 Department of Pediatrics, Division of Allergy and Immunology, Stanford School of Medicine, Stanford, CA, USA; 9 Department of Medicine, Division of Hematology–Oncology, University of California, Los Angeles, Los Angeles, CA, USA; 10 Jonsson Comprehensive Cancer Center at the University of California, Los Angeles, CA, USA; 11 Department of Microbiology and Immunology, University of California, San Francisco, San Francisco, CA, USA; 12 Parker Institute for Cancer Immunotherapy, San Francisco, CA, USA; 13 Swedish Center for Research and Innovation, Swedish Medical Center, Seattle, WA, USA; 14 Providence St. Joseph Health, Renton, WA, USA; 15 Division of Allergy and Infectious Diseases, University of Washington, Seattle, WA, USA

**Keywords:** NKG2A, infectious disease, autoimmune disease, cancer, multi-disease, systems immunology, CD8^+^ T cells, single-cell multi-omics, plasma proteomics

## Abstract

Infection, autoimmunity, and cancer are principal human health challenges of the 21^st^ century. Often regarded as distinct ends of the immunological spectrum, recent studies hint at potential overlap between these diseases. For example, inflammation can be pathogenic in infection and autoimmunity. T resident memory (T_RM_) cells can be beneficial in infection and cancer. However, these findings are limited by size and scope; exact immunological factors shared across diseases remain elusive. Here, we integrate large-scale deeply clinically and biologically phenotyped human cohorts of 526 patients with infection, 162 with lupus, and 11,180 with cancer. We identify an NKG2A^+^ immune bias as associative with protection against disease severity, mortality, and autoimmune/post-acute chronic disease. We reveal that NKG2A^+^ CD8^+^ T cells correlate with reduced inflammation and increased humoral immunity and that they resemble T_RM_ cells. Our results suggest NKG2A^+^ biases as a cross-disease factor of protection, supporting suggestions of immunological overlap between infection, autoimmunity, and cancer.

## Highlights

• NKG2A^+^ bias is associated with reduced acute and post-acute COVID-19 risk
• NKG2A^+^ bias negatively associates with inflammation from viral infection
• NKG2A^+^ bias negatively associates with lupus
• NKG2A^+^ bias is associated with increased survival and immune response in cancer

## Graphical abstract

**Figure:**
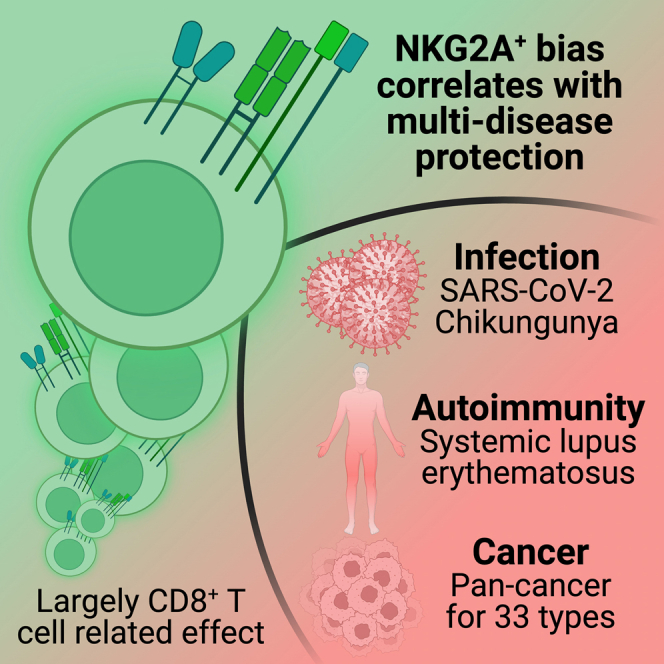

## Teaser

Chen et al. show how an NKG2A^+^-biased immune response associates with biological and clinical correlates of protection and reduced disease severity across multiple cancers, an autoimmune disease (lupus), and multiple viral infections.

## Introduction

Infection, autoimmunity, and cancer are principal drivers of human mortality and disease; they are responsible for over 40% of deaths worldwide.^1^^,^^2^^,^^3^^,^^4^^,^^5^ There is an urgent need to understand shared human immunology across disease contexts to identify potential pan-disease therapeutic strategies. For example, current immunotherapies may provide benefit to the patient for one disease while exacerbating another condition. Specifically, certain cancer immunotherapies, such as immune checkpoint blockade, can promote strong CD8^+^ T cell anti-tumor immunity but can also trigger autoimmunity.^6^ Similarly, many autoimmune disease treatments leverage systemic immunosuppressive drugs that can leave patients more susceptible to infectious diseases and cancer.^7^^,^^8^

It has historically been challenging to execute studies designed to interrogate how immunological factors operate across diverse disease settings, primarily because datasets that match large human clinical cohorts with multi-omic biological data have been limited. However, such studies, even when executed on small patient cohorts representing only a few disease settings, have yielded new insights into T cell biology. For example, one recent study highlighted KIR^+^ CD8^+^ T cells as shared across infection and autoimmunity contexts.^9^ Other studies have connected pathogenic inflammation from both infection and autoimmune disease with antigen-induced cell death (AICD) in cytotoxic T cells.^10^^,^^11^^,^^12^ Further, additional works have established connections between T cell dysfunction in cancer and immune exhaustion in chronic infections such as HIV and hepatitis C virus^13^^,^^14^ and have also revealed T resident memory (T_RM_) cells as essential to sustaining immune responses in both contexts.^15^^,^^16^^,^^17^^,^^18^ While these studies highlight the potential of pan-disease investigations, their limitations in cohort size, number of diseases simultaneously studied, and depth of paired clinical and biological data have made it difficult to identify specific immune factors that can explain shared T cell phenomena across diverse immunological disease settings.

To address this, we home in on NKG2A and NKG2C receptors as putative factors underpinning T cell behaviors across multiple diseases and disease classes. NKG2A/C receptors have recently attracted significant interest within the immunology community, as they allow for T and natural killer (NK) cells to receive potent inhibitory (NKG2A) and stimulatory (NKG2C) signals through non-traditional means (i.e., not through PD1, CD28, etc.). Their presence allows for T and NK cells to be functionally regulated, even when traditional receptors are not present or are blocked. For example, NKG2A can send inhibitory signals via its ITIM (immunoreceptor tyrosine-based inhibitory motif) domains and can disrupt lipid raft formation, potentially disrupting immune synapse signaling.^19^^,^^20^ NKG2C pairs with TYROBP (also known as DAP12) to send ITAM-dependent stimulatory signals.^21^ In addition, there are recent reports that these signals have conserved pan-disease roles in shaping T cell phenotype, such as via chronic antigen stimulation.^6^^,^^22^^,^^23^ Here, we explore the role of NKG2A/C through a systems immunology approach that integrates deep clinical and multi-omic biological data from several large-scale human patient studies covering two infectious diseases, an autoimmune disease (systemic lupus erythematosus [SLE]), and pan-cancer. We find that, in all disease settings, an NKG2A^+^ immune bias, at both bulk and single-cell resolution, associates with decreased patient mortality, decreased severity, and decreased prevalence of autoimmune and post-acute chronic disease. We demonstrate that NKG2A^+^ CD8^+^ T cells significantly associate with protective humoral immunity across diseases and decreased levels of inflammatory cytokines and cell types. We also identify similarities between NGK2A^+^ CD8^+^ T cells and T_RM_ cells in cancerous tumors. In multiple cancer types, we find that these cells correlate with immune infiltration and spatially interact with tumor and immune cells.

## Results

### Multi-omic atlas of NKG2A^+^ and NKG2C^+^ single immune cells and patients from infection, autoimmune, and cancer cohorts

**Figure 1:**
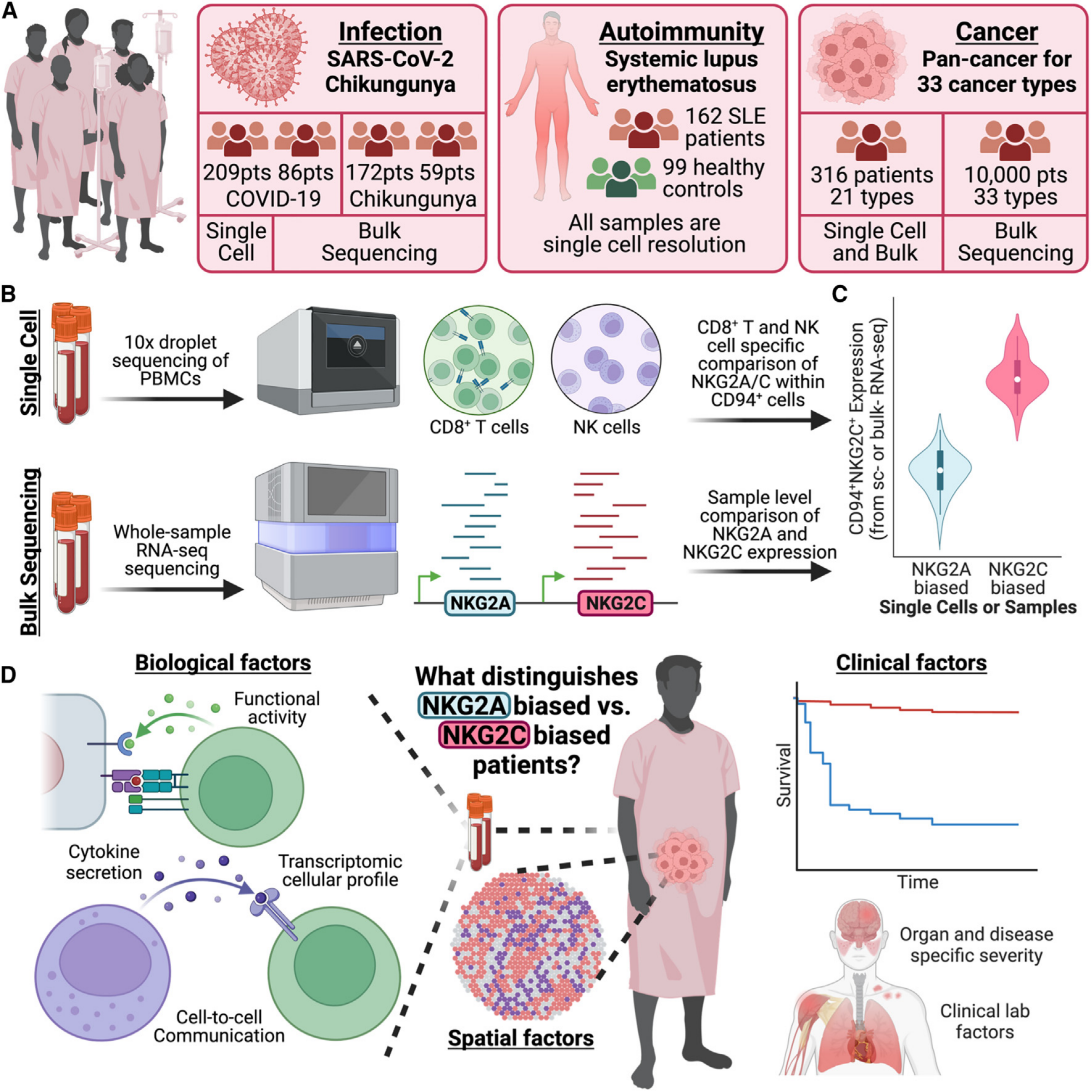
Overview of study design, analytic methods, and dataset multi-omics (A) Cartoon describing the collected single-cell and bulk multi-omic datasets with paired clinical data from infection, autoimmunity, and cancer contexts. (B) Cartoon depicting experimental and analytic strategies for single-cell and bulk data with a focus on NKG2A/C-expressing cell types. (C) Cartoon demonstrating NKG2A^+^ and NKG2C^+^ bias assignment at single-cell and bulk levels. (D) Cartoon displaying the different clinical and biological -omics to be compared between NKG2A^+^ and patients with an NKG2C^+^ bias.

We integrated single-cell and bulk multi-omic datasets from seven highly phenotyped human clinical cohorts for infection (severe acute respiratory syndrome coronavirus 2 [SARS-CoV-2] n = 296 patients and chikungunya n = 231 patients), autoimmunity (SLE n = 162 patients), and pan-cancer (n = 11,180 patients) (Figure 1A; Table S1; see STAR Methods).^24^^,^^25^^,^^26^^,^^27^^,^^28^^,^^29^ As NKG2A/C expression is dominantly restricted to CD8^+^ T cells and NK cells, we focused on these two cell types for single-cell analyses and presumed bulk NKG2A/C measurements to represent expression from these two cell types alone (Figure 1B).^30^ Since NKG2A/C receptors are known to form heterodimers with CD94 for function, we account for CD94 expression when classifying single cells and bulk samples as NKG2A^+^ or NKG2C^+^ biased (Figure 1C; see STAR Methods).^31^^,^^32^ Given the divergent biological signals sent by these two receptors, inhibitory for NKG2A and stimulatory for NKG2C,^32^ and their restriction to T and NK cells,^30^ we hypothesized that NKG2A or NKG2C is likely involved in shared immunopathology and protection factors across disease contexts. Thus, we took on a multi-disease investigation into how NKG2A^+^ and NKG2C^+^ T cell and patient biases associate with biological and clinical markers of disease presence, severity, and patient mortality across three divergent disease states (Figure 1D).

### Patients with infection and NKG2A^+^ bias have decreased disease severity, mortality, and prevalence of post-acute chronic disease

We start by examining patients with infectious diseases, with a focus on our previously reported longitudinal COVID-19 cohort due to its depth of matched clinical and biological profiling.^24^^,^^25^^,^^33^^,^^34^^,^^35^ Consistent with previous works, our assigned NKG2A^+^ and NKG2C^+^ cells presented with divergent phenotypes, with NKG2C^+^ cells appearing more activated in both the CD8^+^ T and NK cell compartments, likely due to the stimulatory nature of the NKG2C receptor (Figure S1A).^31^ Of note, NKG2C^+^ CD8^+^ T cells represented only 1% of total CD8^+^ T cells, suggesting that they are not cytomegalovirus (CMV)-driven populations. NKG2C^+^ CD8^+^ T cells are known to expand to a significantly larger fraction of patient CD8^+^ T cells upon CMV-driven stimulation.^36^ Further, in line with the presence of NK-related NKG2A/C receptors on these unique CD8^+^ T cells, both NKG2A^+^ and NKG2C^+^ cells possessed increased *NCAM1* (CD56) gene expression and surface protein levels compared to other CD8^+^ T cells. CD56 is known to denote NK-like T cells (Figure S1B).^37^^,^^38^^,^^39^^,^^40^ Further, KIR proteins, which have also been reported on NK-like T cells and appear involved in immunoregulatory behaviors,^9^ were markedly upregulated on NKG2A^+^ and especially NKG2C^+^ CD8^+^ T cells (Figure S1C). We see additional confirmation of our NKG2A^+^ and NKG2C^+^ assignment in the NK cell compartment where NKG2C^+^ NK cells indeed present with an adaptive-like phenotype, which NKG2C^+^ NK cells are known to acquire (Figure S1D).^35^^,^^41^ Thus, we confirm the validity of our NKG2A^+^ and NKG2C^+^ cell assignment in all cellular compartments and demonstrate their phenotypic divergence from each other.

**Figure 2:**
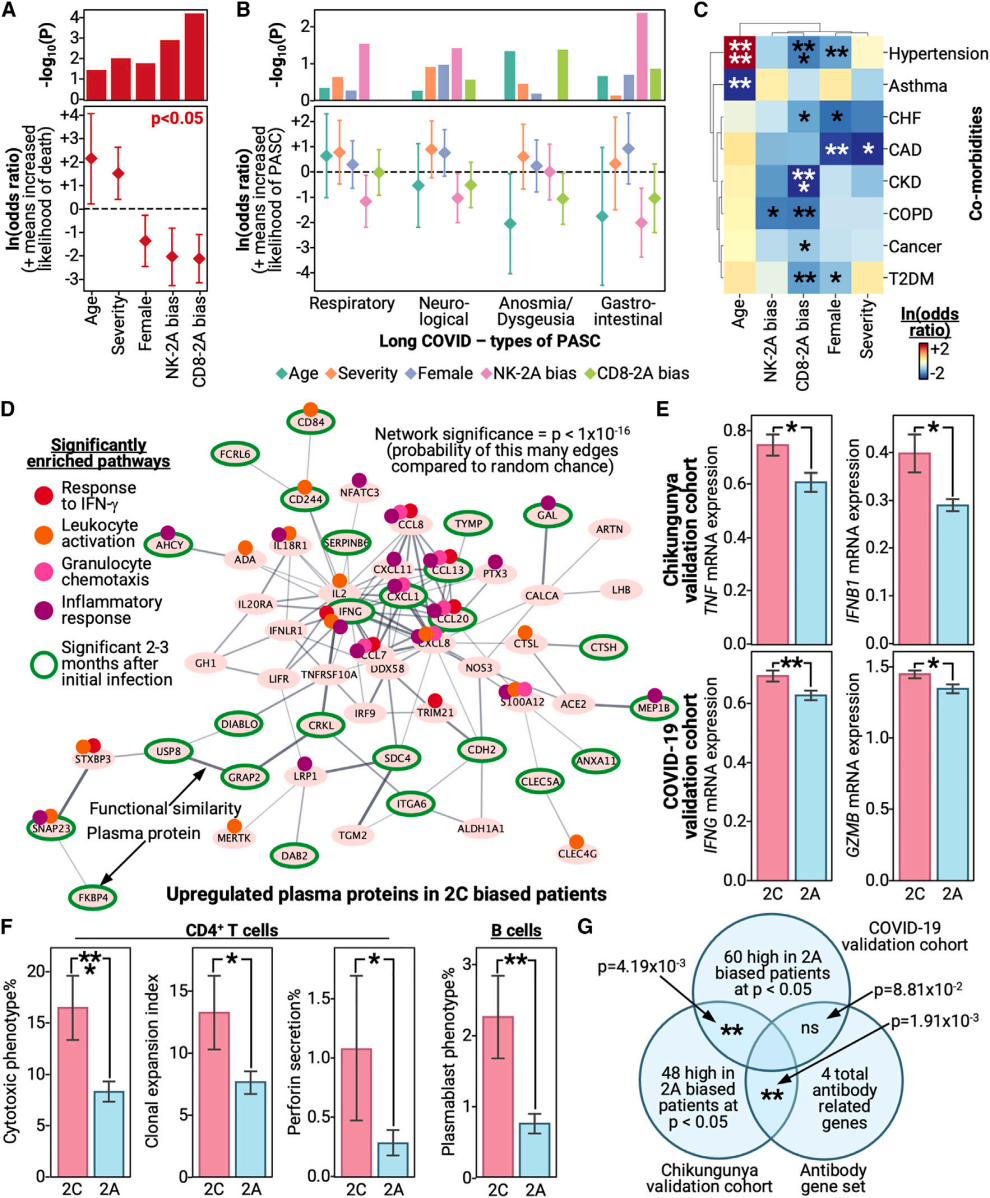
NKG2A^+^ bias associates with clinical and biological correlates of protection in infection contexts (A) Log-odds model of patient mortality predicted by NKG2A^+^ bias while accounting for demographic factors. Top: bar plot with the x axis as different co-variates; the y axis is the −log_10_(p value). Bottom: forest plot with the x axis as different co-variates and the y axis as ln(odds ratio) for a given co-variate, with 95% confidence intervals plotted as whiskers. Red color indicates significance, meaning p < 0.05. (B) Log-odds model of whether a patient has a given long COVID symptom as predicted by NKG2A^+^ bias while accounting for demographic factors. Top: bar plot with the x axis as different co-variates and symptoms; the y axis is the −log_10_(p value). Bottom: forest plot with the x axis as different co-variates and symptoms and the y axis as ln(odds ratio) for a given co-variate, with 95% confidence intervals plotted as whiskers. Colors indicate different co-variates; see legend at bottom. (C) Log-odds model of whether a patient has a given co-morbidity as predicted by NKG2A^+^ bias while accounting for demographic factors. Red color indicates increased ln(odds ratio), as in given the presence of the co-variate on the x axis, there is increased odds of the co-morbidity on the y axis; blue colors indicate a decrease; see legend on bottom right. Co-morbidities are abbreviated as follows: CHF, congestive heart failure; CAD, coronary artery disease; CKD, chronic kidney disease; COPD, chronic obstructive pulmonary disease; T2DM, type II diabetes. (D) Network of plasma proteins upregulated in patients with an NKG2C^+^ bias. Ovals are individual proteins, and line width indicates the strength of their connection; a wider line means increased functional similarity. Significantly enriched pathways are annotated via a smaller circle on the given protein’s oval. Green outlines denote plasma proteins that are significantly upregulated in patients with an NKG2C^+^ bias even 2–3 months after initial infection. (E) Bar plots with upper row from the chikungunya validation cohort and the bottom from the COVID-19 validation cohort. The x axis denotes whether the patient has an NKG2C^+^- (2C) or NKG2A^+^-biased (2A) response, and the y axis represents the mRNA level for the given transcript. (F) Bar plots with measurements from 2 to 3 months after initial infection. The x axis denotes whether the patient has an NKG2C^+^- (2C) or NKG2A^+^-biased (2A) response, and the y axis represents, from left to right, the percentage of CD4^+^ T cells that are cytotoxic from single-cell RNA sequencing (scRNA-seq), the clonal expansion index of CD4^+^ T cells from single-cell T cell receptor sequencing (scTCR-seq), perforin secretion from Isoplexis, and the percentage of B cells that are plasmablasts from scRNA-seq. (G) Venn diagram depicting the overlap between significantly upregulated genes in patients with an NKG2A^+^ bias from the COVID-19 validation cohort (top middle), chikungunya validation cohort (bottom left), and a defined antibody gene set (bottom right). Intersections are annotated with the significance of the overlap. Bar plots are presented as the mean value with standard error. p values are annotated on all relevant plots with either value or stars, ^∗∗∗∗^p < 0.0001, ^∗∗∗^p < 0.001, ^∗∗^p < 0.01, and ^∗^p < 0.05.

To investigate the biological and clinical impacts of an NKG2A/C immune bias during infection, we assigned each patient as NKG2A^+^ or NKG2C^+^ biased based on their ratio of NKG2A^+^ to NKG2C^+^ cells. This assignment was done for each cell type (see STAR Methods). Interestingly, patients with an NKG2A^+^ bias had significantly greater odds of surviving than those with an NKG2C^+^ bias even when accounting for the effects of sex, age, and disease severity (Figure 2A). Similar trends, albeit less significant, associating an NKG2A^+^ bias with increased survival were observed in validation cohorts even when accounting for demographic factors (Figure S2A). Given that expansion of NKG2C^+^ NK cells is associated with prior CMV infection, a known risk factor in infection contexts, we repeated these analyses based on whether a patient had prior CMV infection as a co-variate (see STAR Methods). Interestingly, even when we accounted for prior CMV infection, NKG2A^+^ biases were still significantly associated with greater odds of survival, suggesting that the benefit derived from an NKG2A^+^ bias is not explained by CMV infection history alone (Figure S2B).

To probe for the long-term impact of NKG2A^+^ biases, we compared long COVID profiles of patients who did and did not have NKG2A^+^-biased immune systems during acute disease. Intriguingly, we observed significant protection against long COVID in patients with an initial NKG2A^+^ bias, even when accounting for sex, age, and disease severity (Figure 2B). We found NKG2A^+^-biased NK cells to associate with greater protection than CD8^+^ T cells for all symptom groups except for anosmia/dysgeusia, colloquially called loss of smell/taste. This may be explained by previous observations that anosmia/dysgeusia are driven by fundamentally different pathology than unresolved inflammation,^42^^,^^43^^,^^44^^,^^45^^,^^46^ which associates with other post-acute sequelae. As with the survival analyses, we confirmed that NKG2A^+^ biases, especially those in the NK cell compartment, significantly associate with long COVID protection even when accounting for prior CMV infection (Figure S2C).

The critical role of inflammation in exacerbating severity in acute and post-acute disease suggests that the benefit derived from an NKG2A^+^ bias may in some way be related to quelling inflammation. Consistent with this suggestion, patients with an NKG2A^+^ bias had significantly decreased prevalence of pre-existing chronic conditions even when accounting for demographic differences (Figure 2C). Many of these co-morbidities are known to have inflammation-driven origins or have pathology intimately tied with inflammation, such as chronic obstructive pulmonary disorder and coronary artery disease.^47^^,^^48^ Thus, these findings strongly suggest, across multiple infection contexts and cohorts, that an NKG2A^+^ bias confers protection during acute and post-acute disease given findings of reduced mortality, severity, and long-term symptoms.

### NKG2A^+^ biases correlate with reduced pathogenic inflammation during infection

To investigate the suggestion from clinical data that NKG2A^+^ biases may confer protection through reduced inflammation, we interrogated the biological profiles of patients with an NKG2A^+^ versus an NKG2C^+^ bias for differences in inflammatory proteins and cell types. Consistent with the clinical data, patients with an NKG2A^+^ bias had significantly downregulated levels of inflammatory proteins (Figure 2D; Table S2.1). By contrast, patients with an NKG2C^+^ bias had plasma proteomes enriched for inflammatory pathways, such as interferon γ (IFNγ) response and granulocyte chemotaxis, and upregulated well-known inflammatory proteins, such as IFNγ and CXCL1, even into post-acute disease (Table S2.2).^49^^,^^50^^,^^51^ This elevation of inflammatory proteins in patients with an NKG2C^+^ bias was confirmed in our validation cohorts, suggesting that this association holds across different infection contexts and cohorts (Figure 2E; Tables S2.3–S2.5).

Probing deeper into this connection between inflammation and NKG2A/C biases, we compared the immune cell profiles of patients with NKG2A^+^ biases to those with NKG2C^+^ biases. Even during post-acute disease, patients with an NKG2C^+^ bias presented with signs of continued inflammation as observed through significantly higher levels of cytotoxic CD4^+^ T cells, CD4^+^ T cell clonal expansion, and perforin-secretion capabilities by those cells (Figure 2F). This continued reactivity in the CD4^+^ T cell compartment was accompanied by a simultaneous increase in plasmablast percentages (Figure 2F, right). Given the important collaborative roles of CD4^+^ T and B cells in humoral immunity, we interrogated patients for differences in antibody levels. Notably, while plasmablast levels were upregulated in patients with an NKG2C^+^ bias, it was patients with an NKG2A^+^ bias who had greater odds of developing anti-SARS-CoV-2 antibodies (Figure S3A). In contrast, an NKG2C^+^ bias associated with increased levels of autoantibodies, particularly anti-IFNα2, and the presence of atypical memory (AtM) B cells during acute disease. AtM B cells are often associated with autoantibodies and autoimmunity (Figures S3B and S3C).^52^^,^^53^ This phenomenon of humoral immunity divergence was also observed in the validation cohorts, where we found genes upregulated by patients with an NKG2A^+^ bias in the chikungunya cohort to display significant overlap with antibody-associated genes and nearly significant overlap for the other COVID-19 cohort (Figure 2G). Thus, we demonstrate that, across multiple infection contexts and cohorts, NKG2A^+^ biases not only associate with clinical metrics of protection but also with biological metrics of protection as observed through reduced short- and long-term inflammation, reduced autoreactivity, and increased protective humoral immunity.

### NKG2A^+^ CD8^+^ T cells associate with protection and reduced inflammation in lupus

Signs that NKG2A^+^ biases may potentially associate with protection against autoimmunity pushed us to investigate the role of NKG2A^+^ biases in autoimmune disease. The most well characterized of these is SLE (lupus).^26^^,^^54^ Given recent reports of the importance of CD8^+^ T cells in lupus settings and their ability to carry NKG2A/C receptors, we focused our autoimmune analyses on the clinical and biological impacts of an NKG2A^+^ bias in the CD8^+^ T cell compartment.^55^

**Figure 3:**
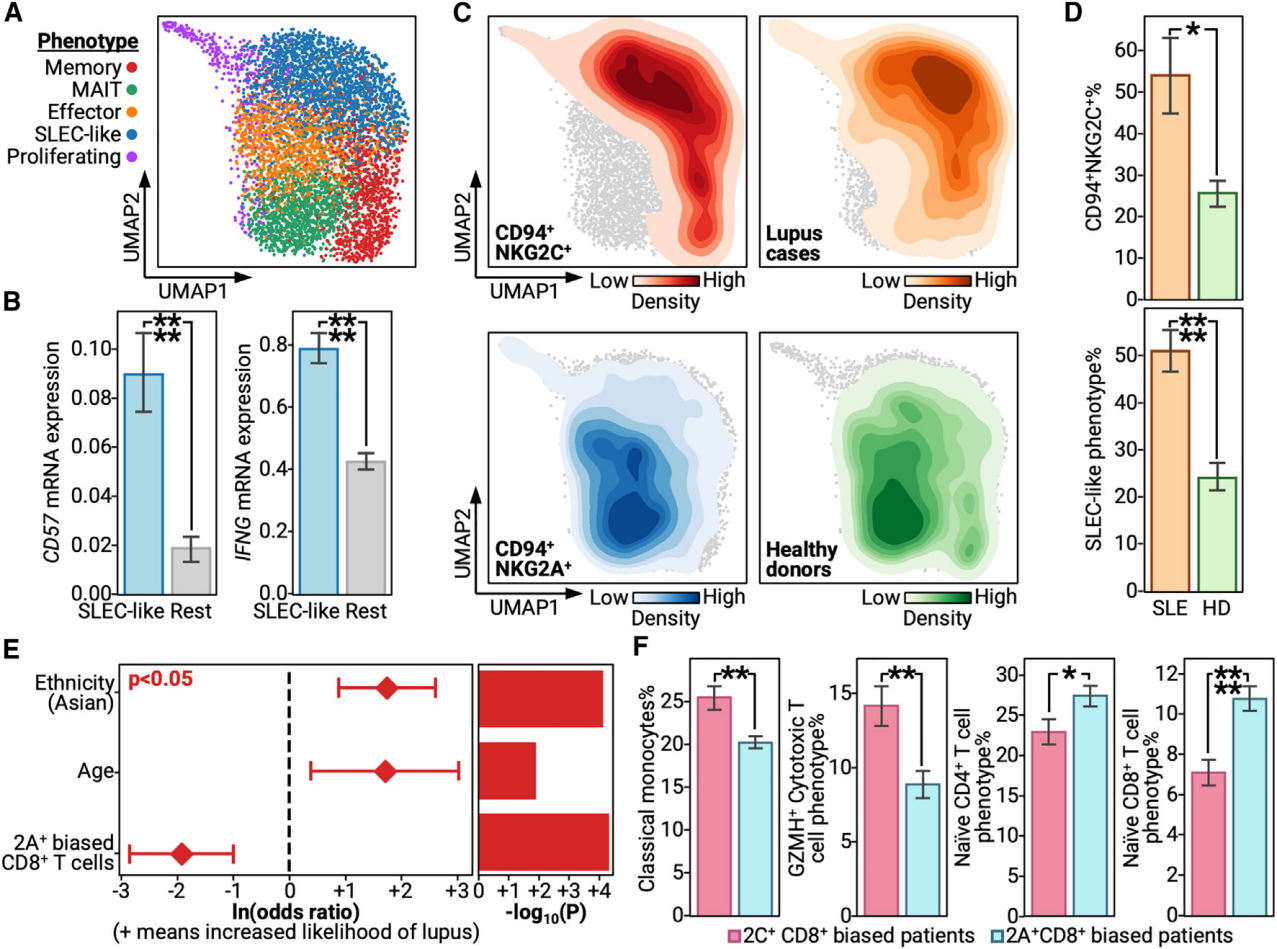
NKG2A^+^-biased CD8^+^ T cells significantly associate with protection from lupus and related inflammation (A) Uniform manifold approximation and projection (UMAP) of single CD8^+^ T cells that are either NKG2A^+^ or NKG2C^+^ from patients with lupus and healthy controls. Colors indicate different phenotypes; see legend on left. Phenotypes are abbreviated as follows: MAIT, mucosal-associated invariant T cells; SLEC, short-lived effector cells. (B) Bar plots with x axis as whether the cell belongs to the SLEC-like phenotype defined in (A), where blue denotes SLEC-like cells and gray denotes other cells, and the y axis as the mRNA level of a given transcript. (C) Density plots on the CD8^+^ T cell UMAP, with darker colors indicating increased density and lighter colors indicating decreased presence. Each color indicates a different cellular or patient subset; see the annotation on the bottom of each UMAP for the legend. (D) Bar plots with x axis as whether the patient has lupus, annotated as SLE with an orange bar, or is a healthy control, annotated as HD with a green bar. The y axis represents the percentage of the given phenotype. (E) Log-odds model of whether a patient has lupus as predicted by prevalence of NKG2A^+^-biased CD8^+^ T cells while accounting for demographic factors. Left: forest plot with the y axis as different co-variates and x axis as ln(odds ratio) for a given co-variate, with 95% confidence intervals plotted as whiskers. Right: bar plot with the y axis as different co-variates; the x axis is the −log_10_(p value). Red color indicates significance, meaning p < 0.05. (F) Bar plots with x axis as whether a patient is biased toward NKG2A^+^ or NKG2C^+^ CD8^+^ T cells, and y axis represents the percentage of a given phenotype out of a given patient’s peripheral blood mononuclear cells (PBMCs). Bar plots are presented as the mean value with standard error. p values are annotated on all relevant plots with either value or stars, ^∗∗∗∗^p < 0.0001, ^∗∗∗^p < 0.001, ^∗∗^p < 0.01, and ^∗^p < 0.05.

NKG2A^+^ and NKG2C^+^ CD8^+^ T cells, when projected onto a transcriptome-defined uniform manifold approximation and projection, present with canonical CD8^+^ T cell phenotypes along with a prominent short-lived effector cell (SLEC)-like population (Figure 3A). SLEC-like cells displayed upregulated levels of CD57, confirming their terminal phenotype, and increased IFNγ mRNA. This suggests that SLEC-like cells may play a role in lupus pathology given previous demonstrations of IFNγ-dependent inflammation and activation of autoreactive cells in patients with lupus (Figure 3B).^56^^,^^57^^,^^58^ When we interrogated SLEC-like cells for NKG2A^+^ or NKG2C^+^ biases, we found a strong NKG2C^+^ bias that was accompanied by a near-complete clinical bias toward patients with lupus (Figure 3C, top ). In contrast, non-SLEC-like cells were nearly all NKG2A^+^ and almost solely derived from healthy donors (Figure 3C, bottom). We statistically confirmed both the increased prevalence of NKG2C^+^ cells in patients with lupus and the lupus-specific CD8^+^ T cell bias toward an SLEC-like phenotype (Figure 3D). Further, we confirmed the SLEC-like phenotype of NKG2C^+^ cells through flow cytometry, where we found NKG2C^+^ cells to present significantly more frequently than NKG2A^+^ cells as CD57^+^IL7R^−^, which is the literature definition of SLECs (Figures S4A–S4C; Table S3).^59^^,^^60^ Thus, we both demonstrate the increased prevalence of NKG2C^+^ CD8^+^ T cells in patients with lupus, but we also experimentally validated their inflammatory SLEC-like phenotype that may, possibly through IFNγ secretion, allow them to play pathogenic roles.

To explore the larger immunological impacts of an NKG2A^+^ bias, we assigned each patient with lupus and each healthy donor as NKG2A^+^ or NKG2C^+^ biased based on their ratio of NKG2A^+^ and NKG2C^+^ CD8^+^ cells (see STAR Methods). Consistent with our CD8^+^ T cell analyses, patients with an NKG2C^+^ bias were more likely to have lupus even when accounting for demographic co-variates (Figure 3E). When we compared the percentages of other immune subtypes against patients with either an NKG2A^+^ or an NKG2C^+^ bias, we observed that an NKG2C^+^ bias associated with a clear increase in inflammatory classical monocytes and cytotoxic T cells. In contrast, an NKG2A^+^ bias associated with increased percentages of naive CD4^+^ and naive CD8^+^ T cells (Figure 3F). These immune cell associations match what was observed for patients in infection contexts. For example, inflammatory cell types are overrepresented in patients with an NKG2C^+^ bias. Thus, we demonstrate that, across disease classes, NKG2A^+^ biases are consistently associated with both clinical protection as well as biological markers of protection, such as reduced inflammation and hyper-activation.

### Patients with cancer and NKG2A^+^ bias have increased survival across cancer types

**Figure 4:**
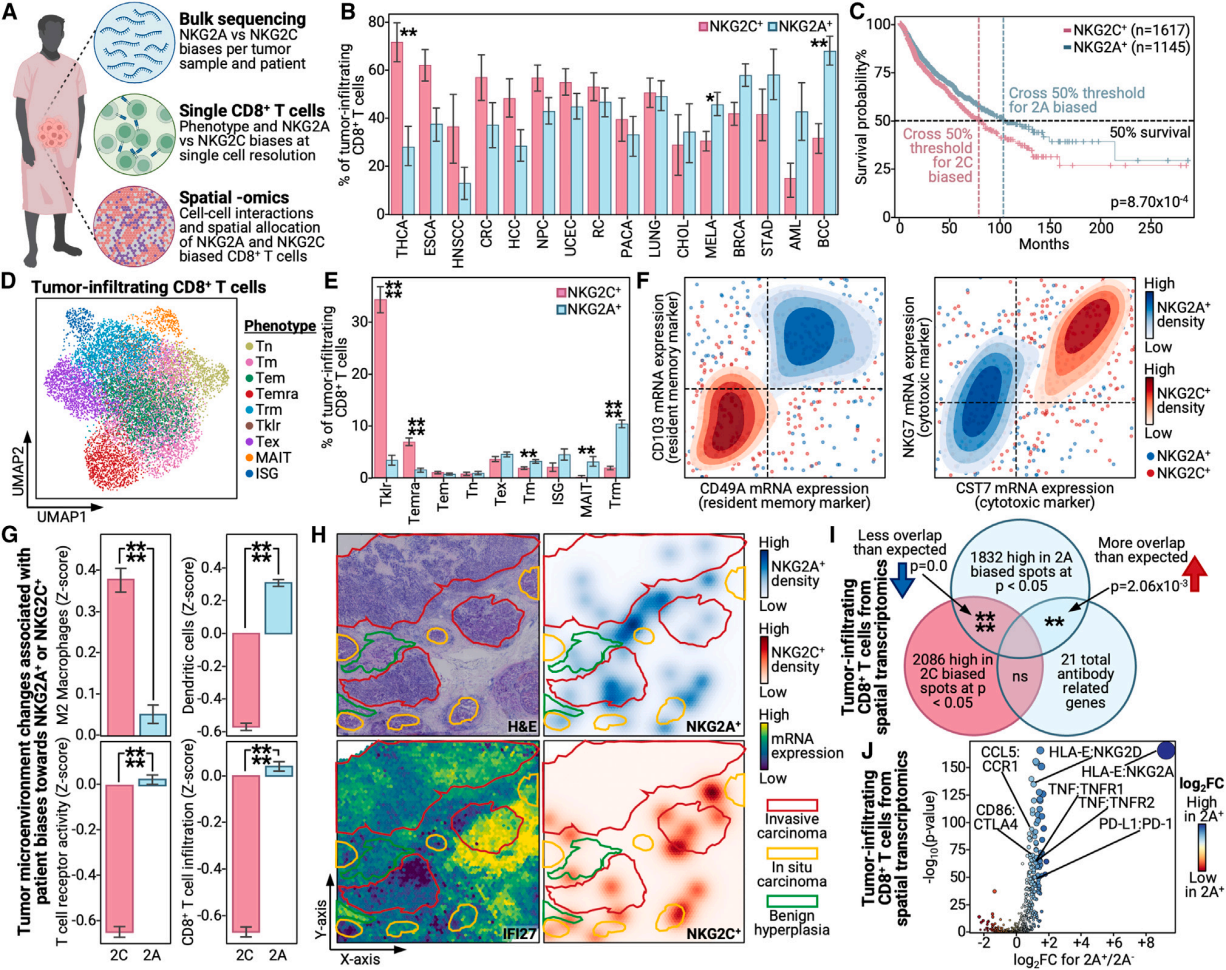
NKG2A^+^ CD8^+^ T cells are present across cancer types and associate with survival and immune infiltration into tumors (A) Cartoon depicting the different biological -omics collected from patients with cancer along with clinical data and the associated analytic methods for each -omic. (B) Bar plots with x axis as different cancer types; the y axis represents the percentage of tumor-infiltrating CD8^+^ T cells for a given cancer type that are NKG2C^+^ (red) and NKG2A^+^ (blue). Cancer types are abbreviated as follows: THCA, thyroid carcinoma; ESCA, esophageal cancer; HNSCC, head and neck squamous cell carcinoma; CRC, colorectal cancer; HCC, hepatocellular carcinoma; NPC, nasopharyngeal carcinoma; UCEC, uterine corpus endometrial carcinoma; RC, renal cancer; PACA, pancreatic cancer; LUNG, lung cancer; CHOL, cholangiocarcinoma; MELA, melanoma; BRCA, breast cancer; STAD, stomach adenocarcinoma; AML, acute myeloid leukemia; BCC, basal cell carcinoma. (C) Kaplan-Meier survival plot of NKG2A^+^- (blue) versus NKG2C^+^ (red)-biased patients based on bulk RNA-seq data from TCGA patients. Log-rank test is used to calculate the displayed p value. (D) UMAP of tumor-infiltrating CD8^+^ T cells from patients across 33 cancer types. Colors indicate different phenotypes; see legend on right. (E) Bar plots with x axis as different cancer types; y axis represents the percentage of tumor-infiltrating CD8^+^ T cells for a given phenotype that are NKG2C^+^ (red) and NKG2A^+^ (blue). (F) Density plots with x and y axes as the mRNA levels for a given transcript. Red densities are for NKG2C^+^ cells and blue densities are for NKG2A^+^ cells; darker colors indicate greater density. Individual data points within the density plot are shown in the same respective colors. See legend on right. (G) Bar plots with x axis indicating whether patients are biased for NKG2C^+^ (red) or NKG2A^+^ (blue) based on bulk data; the y axis represents the *Z* score of a given tumor microenvironment-associated value. (H) Spatial transcriptomics slide for a given patient with breast cancer with H&E (top left), interferon expression (bottom left), NKG2A^+^ cell density (top right), and NKG2C^+^ cell density (bottom right) colored on; see legend on right. Annotations of the slide from pathology are outlined in red for invasive carcinoma, yellow for *in situ* carcinoma, and green for benign hyperplasia. (I) Venn diagram representing the overlap between differentially upregulated genes in spots biased for NKG2A^+^ (top middle), NKG2C^+^ (bottom left), and a defined antibody gene set (bottom right). Significance is indicated in the intersection; a blue down arrow indicates less overlap than expected, and a red up arrow indicates greater overlap than expected. (J) Scatterplot of protein-protein interactions within tumor-infiltrating CD8^+^ T cells with the x axis as the log_2_-transformed fold change (log_2_FC) of NKG2A^+^ CD8^+^ T cell^+^ spots over other CD8^+^ T cell spots, and the y axis is the −log_10_(p value). Color corresponds to log_2_FC, see legend on right, and dot size is the product of the x and y values. Individual interactions with clinical and biological relevance are labeled. Bar plots are presented as the mean value with standard error. p values are annotated on all relevant plots with either value or stars, ^∗∗∗∗^p < 0.0001, ^∗∗∗^p < 0.001, ^∗∗^p < 0.01, and ^∗^p < 0.05.

While we observed NKG2A^+^ biases as associated with protection within the context of two viral infections and one autoimmune disease, these are diseases where inflammation is closely tied to, if not the source of, the disease pathology. Thus, an NKG2A^+^ bias is understandably protective, as it provides cells an additional method to receive inflammation quelling signals. However, in cancer contexts, inflammation has been claimed as both beneficial and harmful: beneficial for the promotion of immune activation and infiltration and harmful due to inflammation- and activation-induced apoptosis of anti-tumor immune cells.^11^^,^^61^^,^^62^^,^^63^ Thus, the impact of NKG2A^+^ biases in cancer contexts is unclear. To address this, we compiled single-cell and bulk profiles of 397,810 tumor-infiltrating CD8^+^ T cells and 11,180 patients with cancer across 33 different cancer types (Figure 4A).^28^^,^^29^ In addition, we gathered spatial transcriptomics data from five different patients for breast, prostate, and ovarian cancer to understand how cell-cell interactions differ between NKG2A^+^ and NKG2C^+^ CD8^+^ T cells across cancer types.^64^

To assess the prevalence of NKG2A^+^ and NKG2C^+^ biases across different cancer types, we measured the percentages of NKG2A^+^ and NKG2C^+^ CD8^+^ T cells in cancer types for which we had large numbers of patients with single-cell data. Interestingly, not only did we observe widespread prevalence of these immune cell subsets among cancer types, but cancer types differed in their tendency toward an NKG2A^+^- or NKG2C^+^-biased response (Figure 4B). Interestingly, melanoma, which is well known for fostering an immunogenic tumor microenvironment (TME),^65^ appeared significantly NKG2A^+^ biased. In contrast, thyroid cancer, which presents as a cold tumor with few immunogenic antigens,^66^ was significantly NKG2C^+^ biased. In line with these observations, we found NKG2A^+^-biased cancer types to mildly associate with greater tumor immunogenicity (Figure S5A). This suggestion of NKG2A^+^ association with pro-immune response TMEs prompted us to compare the long-term survival of patients with NKG2A^+^- versus NKG2C^+^-biased tumors. Interestingly, NKG2A^+^-biased tumors associated with significantly increased pan-cancer patient survival rates, with especially prominent survival benefits for specific cancers, such as certain renal cancer subtypes (Figures 4C and S5B). For no cancers did an NKG2C^+^ bias confer a survival advantage. Thus, across a broad range of cancer types, we find that NKG2A^+^ biases are positively associated with cancer survival. In contrast to previous suggestions that NKG2A may function as a druggable immune checkpoint,^23^ our analyses suggest that, instead, treatments designed to promote NKG2A^+^-biased immune responses may benefit patients with cancer.

### NKG2A^+^ CD8^+^ T cells in tumors associate with increased immune infiltration and tumor-immune interactions

To more deeply investigate the biological underpinnings behind the seemingly beneficial NKG2A^+^ bias, we sought to understand how NKG2A^+^ CD8^+^ T cell tumor infiltrates differ from their NKG2C^+^ counterparts. To achieve this, we examined the transcriptomic profiles of nearly 400,000 CD8^+^ T cells from 21 cancer types (Figure 4D) and assigned them as NKG2A^+^, NKG2C^+^, or neither based on mRNA expression levels (see STAR Methods). Interestingly, we observed NKG2A^+^ and NKG2C^+^ cells to occupy distinct phenotypes with NKG2A^+^ cells significantly biased for a T_RM_ phenotype and NKG2C^+^ cells biased toward T_EMRA_ and killer cell lectin-like receptor-expressing T cell phenotypes (Figure 4E).^67^^,^^68^ The preference of NKG2A^+^ cells toward a T_RM_ phenotype and NKG2C^+^ cells toward more strongly activated phenotypes was confirmed through their differential expression of key marker genes (Figure 4F; Table S4). T_RM_ cells have been well characterized as potent supporters of anti-infection and cancer immune responses, and their presence is associated with survival. T_RM_ cells are also known to present with an activated phenotype that allows for effector responses while avoiding AICD.^15^^,^^18^ These characterizations not only match the observed benefit NKG2A^+^ biases provide patients with cancer but also align with clinical and biological characterizations of NKG2A^+^ CD8^+^ T cells and biases we observed in infection and autoimmunity contexts. This suggests that the pan-disease benefit of NKG2A may arise from NKG2A^+^ cells biasing toward T_RM_ or analogous phenotypes that allow for control of a given target without pathogenic inflammation.

To further characterize the biological nature of an NKG2A^+^ bias in cancer settings, we compared the TME profiles of patients with NKG2A^+^ and NKG2C^+^ biases. In agreement with our previous suggestions of increased immune activity in NKG2A^+^-biased tumors, patients with NKG2A^+^ biases presented with increased CD8^+^ T cell infiltration, T cell receptor engagement, dendritic cell presence, and decreased prevalence of M2 macrophages (Figure 4G). The first three factors not only confirm that patients with an NKG2A^+^ bias have increased immune infiltration in their tumors but also suggest that patients with an NKG2A^+^ bias are equipped with the proper immune machinery of dendritic cells to prime and present CD8^+^ T cells with tumor antigens to permit cancer cell recognition and killing. M2 macrophages are well known to be pro-tumorigenic, have been frequently associated with metastasis, and are claimed as direct players in fostering cold immunosuppressive TMEs.^69^^,^^70^^,^^71^^,^^72^^,^^73^ Thus, their decreased prevalence in patients with NKG2A^+^ biases demonstrates that NKG2A^+^ biases not only associate with increased immune infiltration of key anti-tumor players but also positively correlate with immune cell behaviors that facilitate, not hinder, anti-tumor responses.

Inspired by these suggestions of NKG2A^+^ biases fostering pro-survival anti-cancer TMEs, we gathered spatial transcriptomics samples from five separate patients that comprised three different cancer types: breast, prostate, and ovarian (Figure 4A).^64^ One of the breast cancer samples was already well characterized by a pathologist, and thus we focused our analyses on that sample. Reminiscent of their divergent phenotypes, NKG2A^+^ and NKG2C^+^ spots occupied distinct spatial regions of the tumor (Figure 4H). While NKG2C^+^ spots did differ from NKG2A^+^ spots by sitting in an IFN-expressing zone of the tumor, both subsets were either in or directly adjacent to tumor tissue, perhaps suggesting active tumor engagement and possibly killing. Further, consistent with the observed association between NKG2A^+^ biases and immune infiltration, differentially upregulated genes in NKG2A^+^ spots were significantly enriched for antibody-related genes (Figure 4I). This phenomenon matches our observations across viral infection contexts and thus suggests that this association may hold true across very diverse disease settings. To confirm the active immune cell and tumor engagement suggested to occur in NKG2A^+^ spots, we performed cell-cell interaction analysis by looking for ligand-receptor pairs differentially enriched in NKG2A^+^ CD8^+^ T cell spots compared to NKG2A^−^ CD8^+^ T cell spots across all five spatial transcriptomics datasets (Figure 4J). Interestingly, not only did we observe enrichment for HLA-E:NKG2A, which suggests that there may be active engagement of NKG2A in tumors, but we also observed increased chemokine, cytokine, and exhaustion receptor engagement. This suggests active recruitment of immune cells, consistent with increased immune infiltration in bulk RNA sequencing samples, and suggests the possibility of tumor-immune cell engagement, for example through the well-known PD-1:PD-L1 axis.^74^^,^^75^^,^^76^ Thus, we demonstrate across dozens of cancer types, thousands of patients, and hundreds of thousands of tumor-infiltrating CD8^+^ T cells that NKG2A^+^ biases associate with increased survival and immune infiltration of tumors, as confirmed through *in situ* measurements, possibly due to the acquirement of a T_RM_ phenotype by NKG2A^+^ CD8^+^ T cells that allows for sustained anti-tumor effector T cell responses.

## Discussion

Therapies that target disease specific biology often fail to account for the negative impacts of said therapy in different health contexts. This can subject patients to harmful side effects, as patients can transition in and out of different health states even during the same disease journey. Current immunotherapies often have this pitfall of singular-context benefit. One prime example is immune checkpoint blockade cancer immunotherapy and its established potential to trigger immune-related adverse events (AEs), many of which are largely autoimmune in nature.^62^^,^^77^ Similar dilemmas arise for autoimmune disease, where clinicians must balance the potential benefit of immune-suppressive treatments, such as corticosteroids or anti-interleukins, against the known increased risk of infection.^8^^,^^78^ These principal examples highlight the cross-field need to identify fundamental immunology shared across diseases to identify immunological factors that, when promoted or avoided, allow for a balanced immune response that can clear pathogenic elements without immune-related side effects.

Here, we leverage systems biology techniques to integrate immunological cell types across multiple disease states to demonstrate that an NKG2A immune bias, and thus an increased presence of NKG2A^+^ CD8^+^ T cells, serves as a cross-disease correlate of clinical and biological protection. This shared benefit across multiple infectious diseases, at least one autoimmune disease, and many cancer types reinforces recent suggestions of significant similarities across these disease families.^6^ By interrogating the biological associations of NKG2A^+^ biases, we suggest that hyper-inflammation is pathogenic in infection, autoimmune, and cancer contexts. In fact, our findings suggest that the promotion of non-hyper-inflammatory NKG2A^+^ cells may achieve the desired goals of a sustained anti-tumor immune response: an orchestrated immune infiltration into the tumor and improved patient survival. Such benefits may be attributable to the T_RM_ phenotype that NKG2A^+^ CD8^+^ T cells dominantly bear. T_RM_ cells are thought to play an immunological balancing act where they are restrained enough to avoid AICD yet activated enough that they can enact potent anti-tumor immune responses.^16^^,^^18^ Further, both T_RM_ cells and NKG2A expression can be induced through interleukin-12 (IL-12) and transforming growth factor β (TGF-β), a unique combination of cytokines that confers both effector- and memory-related responses and may allow for the long-term survival and effector function of these cells.^79^^,^^80^ In support of our suggestion that NKG2A^+^ cells present as protective, recent checkpoint blockade clinical trials targeting NKG2A have had markedly limited success and report a significant number of treatment-emergent serious AEs, likely due to the inhibition of inflammation-quelling signaling from NKG2A engagement.^81^

### Limitations of the study

Our multi-disease analysis of NKG2A^+^ cells, while principally a correlative study, also suggests a roadmap for future mechanistic and causal studies that might more clearly resolve how an NKG2A bias associates with immunological protection in multiple disease settings. Dissecting the biological mechanisms behind NKG2A signaling, perhaps through quantitative epigenetic analyses designed to identify signaling and transcription factors that control the up- and downstream regulators of NKG2A^+^ cells, would constitute an important first step and may shed light on perturbations that can alter NKG2A expression levels. An additional avenue of exploration will be to more fully resolve the strong association reported here between NKG2A and resident memory T cells and also to observe whether, and if so how, NKG2A biases change over patient disease journeys. This work also serves to highlight the value of cross-disease, systems biology studies that can quantitate immunological signatures in multiple contexts. We show that such an approach can not only resolve robust correlates of protection but may also force a rethinking of what does and does not constitute a good drug target for immunotherapy. It is worth noting that the NKG2A^+^-biased response we observe here for NK cells is limited to responses that do not require a NKG2C^+^ NK cell response. Our levels of NKG2C^+^ NK cells are much lower than what one would expect for an NKG2C^+^-inducing infection, such as by CMV; thus, they likely represent baseline levels, such as baseline inflammation, rather than the capacity to respond to, for example, Epstein-Barr virus (EBV), whose control by NKG2C^+^ NK cells has been linked to reduced prevalence of multiple sclerosis.^82^

## STAR★Methods

### Key resources table

**Table:** 

REAGENT or RESOURCE	SOURCE	IDENTIFIER
**Antibodies**		

APC anti-human CD57 Antibody	BioLegend	Cat#359609; RRID:AB_2562756
FITC anti-human CD56 (NCAM) Antibody	BioLegend	Cat#318303; RRID:AB_604091
BD OptiBuild BUV395 anti-human CD8	BD Biosciences	Cat#740303; RRID:AB_2740042
Brilliant Violet 421 anti-human CD159a (NKG2A) antibody	BioLegend	Cat#375139; RRID:AB_2941547
PE anti-human CD159c (NKG2C) Antibody	BioLegend	Cat#375003; RRID:AB_2888871
APC/Cyanine7 anti-human CD127 (IL-7Rα) Antibody	BioLegend	Cat#351347; RRID:AB_2629571
Brilliant Violet 785 anti-mouse/human KLRG1 (MAFA) antibody	BioLegend	Cat#138429; RRID:AB_2629749
PE/Cyanine7 anti-human CD16 antibody	BioLegend	Cat#302015; RRID:AB_314215
LIVE/DEAD Fixable Aqua Dead Cell Stain Kit, for 405 nm excitation	ThermoFisher	Cat#L34965

**Biological samples**		

Samples from patients with lupus (human UNCOVR cohort)	Swedish Medical Center; Providence St. Joseph Health; Institute of Systems Biology	N/A
Samples from healthy donors	Bloodworks Northwest	N/A
Samples from healthy donors	Stemcell	Cat#200-0092

**Deposited data**		

COVID-19 single cell cohort (Seattle)	Su et al.^24^ Su et al.^25^	E-MTAB-10129 E-MTAB-9357
COVID-19 cohort (Greece)	Zheng et al. 2^27^	inflammatix86
Chikungunya cohort (adult)	Zheng et al.^27^ Soares-Schanoski et al.^83^	PRJNA507472
Chikungunya cohort (pediatric)	Zheng et al.^27^ Michlmayr et al.^84^	PRJNA390289
Lupus single cell cohort	Perez et al.^26^	GSE174188
Pan-cancer single cell cohort	Zheng et al.^28^	GSE156728
Pan-cancer TCGA cohort	Thorsson et al.^29^	https://portal.gdc.cancer.gov/
Spatial cancer cohort	10x Genomics	10x Visium (v1) spaceranger count datasets for breast, prostate, and ovarian

**Other**		

Scanpy (v1.9.3)	Wolf et al.^85^	https://github.com/scverse/scanpy
harmonypy (v0.0.9)	Korsunsky et al.^86^	https://github.com/slowkow/harmonypy
bbkNN (v1.5.1)	Polański et al.^87^	https://github.com/Teichlab/bbknn
UMAP (v0.5.3)	McInnes et al.^88^	https://github.com/lmcinnes/umap
Leiden (v0.9.1)	Traag et al.^89^	https://github.com/vtraag/leidenalg
STRING (v12)	Szklarczyk et al.^90^	https://string-db.org/
BayesSpace (v1.1.3)	Zhao et al.^64^	https://www.bioconductor.org/packages/release/bioc/html/BayesSpace.html
statsmodels (v0.13.2)	Seabold and Perktold et al.^91^	https://www.statsmodels.org
scipy (v1.9.3)	Virtanen et al.^92^	https://scipy.org/
cellphoneDB (v4)	Garcia-Alonso et al.^93^	https://www.cellphonedb.org/
FlowJo (v10.9.0)	BD Life Sciences	https://www.flowjo.com/

### Resource availability

#### Lead contact

Further information and requests for resources and reagents should be directed to and will be fulfilled by the lead contact, James R. Heath (jim.heath@isbscience.org).

#### Materials availability

This study did not generate new unique reagents.

#### Data and code availability

• For infectious disease: COVID-19 single cell data were retrieved from our previously published multi-omics dataset,^24^^,^^25^ COVID-19 validation cohort and chikungunya adult and pediatric validation cohorts were kindly provided by Dr. Purvesh Khatri and his team from "inflammatix86" (COVID-19 cohort from Greece), "PRJNA507472", and "PRJNA390289"; these datasets were also previously published and analyzed by them.^27^^,^^83^^,^^84^
• For autoimmune disease: systemic lupus erythematosus single cell data were retrieved from Perez et al., 2022.^26^
• For cancer: pan-cancer tumor-infiltrating single CD8^+^ T cell data were retrieved from Zheng et al., 2021, TCGA data are from Thorsson et al., 2018 retrieved via NCI-GDC.^28^^,^^29^ Spatial datasets were retrieved from Zhao et al., 2021 and 10x Genomics.^64^
• This paper does not report original code. Scripts were run using public Python and R packages and are available upon reasonable request.
• Any additional information required to reanalyze the data reported in this work is available upon reasonable request.

### Experimental model and study participant details

For infectious disease: COVID-19 single cell data were retrieved from our previously published multi-omics dataset.^24^^,^^25^ We utilized the dataset as was published, an H5AD file, and filtered for NKG2A/C positive cells, see next section. COVID-19 validation cohort and chikungunya adult and pediatric validation cohorts, bulkRNA-seq, were kindly provided by Dr. Purvesh Khatri and his team with both raw and normalized gene expression values, we only utilized the normalized values. They were formally named the following "inflammatix86" (COVID-19 bulkRNA-seq from Greece), and "PRJNA507472" and "PRJNA390289" (chikungunya bulkRNA-seq datasets) and were previously utilized and published.^27^^,^^83^^,^^84^ For autoimmune disease: systemic lupus erythematosus single cell data were retrieved from Perez et al., 2022,^26^ we utilized ln(CPM +1) normalized values for our analyses. For cancer: pan-cancer tumor-infiltrating single CD8^+^ T cell data were retrieved from Zheng et al., 2021, this was normalized by the previous authors via library size and a per-gene *Z* score, and we retrieved TCGA data from Thorsson et al., 2018 via NCI-GDC via controlled access requests.^28^^,^^29^ Spatial datasets were retrieved from Zhao et al., 2021 and publicly available 10x Genomics datasets.^64^ They were normalized and processed using BayesSpace (v1.1.3). Aside from the spatial workups, Scanpy (v1.9.3) was utilized for the vast majority of these analyses, additional packages and their versions are listed in their appropriate sections. Four lupus patients enrolled in a control arm of a study of long COVID (UNCOVR, Providence St. Joseph Health IRB: STUDY2020000852) contributed PBMCs for comparisons with Bloodworks and Stemcell acquired controls.

### Method details

#### Log-odds models of NKG2A^+^ biases accounting for demographic factors

Log-odds was modeled using the Logit modeling function within the statsmodels (v0.13.2) package. Sex was binarized and people with a female sex were given the value one while those with a male sex were given the value 0. Age was accounted for in years. If NKG2A positivity was not already called at a sample level (i.e., single-cell datasets) then an NKG2A^+^ bias was called for a given patient if they had three times more NKG2A^+^ cells than NKG2C^+^ cells for NK cells, due to the inherent NKG2A^+^ bias in NK cells, and if they had more NKG2A^+^ cells than NKG2C^+^ cells for CD8^+^ T cells. For the COVID-19 patient dataset, as we had multiple timepoints during acute infection, NKG2A^+^ bias was assigned if the patient had a bias at any of the acute timepoints. Further, severity was determined as WHO Ordinal Scale (WOS) value greater than or equal to five, a previously determined value for severe patients.^24^ For lupus patients, the relative ratio of NKG2A^+^ and NKG2C^+^ CD8^+^ T cells was utilized. For validation cohorts, any demographic factors were utilized when available, this mainly consisted of a patient’s assigned sex. All coefficient estimates were plotted with 95% confidence intervals.

#### HCMV serostatus

To predict CMV serostatus of patients from immunosequencing data, we replicated a previously published classification model from Emerson et al., 2017.^94^ Using the study’s two cohorts (HIP and KECK) and its list of 164 CMV-associated TCRβ chains (defined by CDR3 amino acid sequence, V gene, and J gene), we trained and validated our classifier that predicted CMV serostatus using the number of detected CMV^−^ associated TCRβs and the total number of unique TCRβs. The classifier model was trained using a support vector machine with linear kernel and 6-fold cross validation. Based on area under the receiver operating characteristic curve (AUROC), the performance of this classifier was during training. The best performing model was used to predict CMV serostatus of the validation cohort (AUROC = 0.92) and of our INCOV cohort. Patients with any samples predicted to be CMV positive are labeled as CMV positive. Convalescent serum sample from predicted CMV^+^ patients were used for CMV viremia and CMV serology assays. 75/82 (91%) of these samples had positive results from CMV serology assays. We then utilized these CMV serostatus values to account for prior CMV infection history in our log-odds models by adding it as an additional co-variate.

#### Enrichment analysis of NKG2C^+^ enriched plasma proteome

Differentially expressed proteins, as identified across all three measured timepoints at p < 0.05 and greater than zero log-transformed fold change, were compiled and pathways were enriched for via the multiple protein enrichment analysis by the STRING database.^90^

#### Hypergeometric overlap probability analysis

Hypergeometric overlap probabilities were calculated using scipy (v1.9.3) using the cumulative density function (CDF). The p value for testing the hypothesis that two sets overlap as much as is measured or more, is then defined as 1 – CDF, and therefore the probability that two sets overlap less than expected is defined as the CDF itself.

#### CD8^+^ T cell phenotype assignment in lupus patients

NKG2A/C^+^ CD8^+^ T cells were extracted based on the assignments aforementioned in the “NKG2A^+^ and NKG2C^+^ quantification and assignment” section in the Methods. Genes with zero expression within the NKG2A/C^+^ CD8^+^ T cell subset were removed. Highly variable genes were called using the “Seurat” method with the minimum dispersion of 0.5 and mean between 0.5 and 7.5 to remove constantly lowly and constantly highly expressed genes. Principal component analysis (PCA) was then called and 20 PCs were then utilized to compute uniform manifold approximation projections (UMAP via umap-learn v0.5.3) using a k-nearest-neighbor (kNN) graph computed via bbkNN (v1.5.1).^87^^,^^88^ Leiden clusters were then called via the leidnalg package (v0.9.1) with resolution of 0.4. Clusters were then annotated via literature derived genes: proliferation via MKI67, MAIT via KLRB1, memory via IL7R, short lived effector cell (SLEC) -like via CD57 and IFNγ, and effector as non SLEC-like but still expressing GZMB.^63^^,^^95^^,^^96^^,^^97^

#### Flow cytometry validation of NKG2A/C phenotypes

Four PBMC vials from patients with lupus were taken from our UNCOVR database, and four healthy donors, from Bloodworks and Stemcell, were taken as well. These samples were assayed with the following antibodies: APC anti-human CD57 Antibody (BioLegend #359609), FITC anti-human CD56 (NCAM) Antibody (BioLegend #318303), BD OptiBuild BUV395 anti-human CD8 (BD Biosciences #740303), Brilliant Violet 421 anti-human CD159a (NKG2A) antibody (BioLegend #375139), PE anti-human CD159c (NKG2C) Antibody (BioLegend #375003), APC/Cyanine7 anti-human CD127 (IL-7Rα) Antibody (BioLegend #351347), Brilliant Violet 785 anti-mouse/human KLRG1 (MAFA) antibody (BioLegend #138429), PE/Cyanine7 anti-human CD16 antibody (BioLegend #302015), and LIVE/DEAD Fixable Aqua Dead Cell Stain Kit, for 405 nm excitation (ThermoFisher #L34965). PBMC samples were resuspend from −80°C in R10 media to remove and dilute freezing media. Post-washing each pellet was resuspended and washed in one mL ice-cold PBS then resuspended in live-dead stain for 30 min at 4°C. Stained cells were then washed in one mL ice-cold PBS and stained with the surface-protein antibody master mix for 30 min at 4°C. Stained cells were then washed with cell staining buffer, the same that was utilized for the master mix, and resuspended in 100 μL and run on BD FACSymphony A5 Cell Analyzer at Fred Hutchinson Cancer Center.

#### Tumor immunogenicity and NKG2A/C percentage analysis

Cancer types were ranked by a previously published paper that took tumor mutational burden and antigen presentation machinery into account.^66^ Spearman correlation was used because it is more suited for rank-based analyses compared to Pearson correlation which is best suited for continuous against continuous correlations.

#### Kaplan-meier survival analysis on TCGA samples

TCGA samples were called as NKG2A^+^ or NKG2C^+^ via the aforementioned log_2_ fold change criteria. To ensure we only utilized confidently assigned patients, we took only the top 20% and bottom 20%, similar to the aforementioned bulkRNA-seq analyses above. Kaplan-meier curves were then called for each cancer type individually and then for all cancer types and patients together as a “pan-cancer” model.

#### Breast cancer spatial transcriptomics characterization

All spatial datasets were downloaded from 10x Genomics’ publicly available datasets and processed via BayesSpace as aforementioned. Pathology annotations were taken directly from those done in Zhao et al., 2021.^64^ Density plots for NKG2A/C positive cells were computed by utilizing spatial neighbors; each positive spot was diffused outwards to determine regions of occupancy for NKG2A^+^ or NKG2C^+^ spots. Denser regions, the densest being the positive spots themselves, are plotted as darker colors while less dense regions are plotted with lighter colors. This method is the spatially analogous method to those typically done to compute embedding density for transcriptomic or multi-omic UMAPs (e.g., those via scanpy.tl.embedding_density). Hypergeometric analysis was done as stated in previous Methods sections. For hypergeometric tests that involve antibodies, both those for this spatial transcriptomics cancer dataset, and for the infectious disease datasets, genes were considered if they were confidently expressed; defined as within the top 10% for the infection contexts and greater than 0.01 for genes in the cancer context. The antibody gene set was defined as those from heavy chain immunoglobin genes, and light chain lambda and kappa immunoglobin genes.

#### Spatially-informed cell-cell ligand-receptor analysis

All spatial transcriptomics datasets were concatenated together using Scanpy and AnnData objects. Identified NKG2A^+^ and NKG2A^−^ CD8^+^ T cells were called via the Methods in the aforementioned sections and ligand-receptor interactions were called from the CellphoneDB list of known protein-protein interactions through a product calculation.^93^ Mann-Whitney U-tests were then utilized to calculate differential protein-protein interaction values between NKG2A^+^ and NKG2A^−^ CD8^+^ T cell subsets.

### Quantification and statistical analysis

#### NKG2A^+^ and NKG2C^+^ quantification and assignment

For single cell datasets: where log-transformed library-normalized values were available, for example ln(CPM+1), we called cells NKG2A^+^ if they had mRNA levels of CD94 and NKG2A that were ≥2.5. Similarly, NKG2C^+^ cells were called if they had CD94 and NKG2C mRNA levels that were ≥2.5. We require the simultaneous expression of CD94 when it is feasibly measured and analyzed because it forms a hetero-dimer with NKG2A/C to permit their expression.^31^ For Zheng et al.’s dataset, as it was *Z* score normalized absolute expression based analyses were not feasible. Thus, we derived an NKG2A/C metric based on the relative NKG2A to NKG2C Z-scores, those who NKG2A value was at least one greater than their NKG2C value were deemed NKG2A^+^. Similarly, cells with NKG2C values at least one greater than their NKG2A values were deemed NKG2C^+^. For bulk datasets: log_2_ fold changes were calculated between the product of a given samples CD94 and NKG2A (or NKG2C) normalized expression levels. To analyze confidently assigned NKG2A^+^ and NKG2C^+^ cells we deemed NKG2A^+^ samples as those in the upper pentile (top 20%) of log_2_(CD94:NKG2A/CD94:NKG2C) patients, and similarly NKG2C^+^ samples were those in the lowest pentile (bottom 20%). For spatial datasets: due to the sparse nature of the captured expression, for the breast cancer sample, those with NKG2A or NKG2C expression were deemed NKG2A^+^ or NKG2C^+^, respectively. When combining all of the spatial transcriptomic datasets together we were able to more robustly identify CD8^+^ T cells, using CD3 and CD8 mRNA expression, and within that subset classified cells as NKG2A^+^ or NKG2A^−^ based on their expression. Specifically, a T cell score was determined through the average expression of *CD3D*, *CD3E*, and *CD3G*. Cells positive for this score were then interrogated for CD8 expression via averaging of *CD8A* and *CD8B* mRNA. Cells positive for this score were now considered CD8^+^ T cells, we then simply utilized NKG2A expression to divide CD8^+^ T cells into an NKG2A^+^ CD8^+^ T cell and NKG2A^−^ CD8^+^ T cell subset.

#### Statistical analyses

All correlations were calculated using Pearson, and all p values were calculated using Mann-Whitney U test unless otherwise specified. Log-odd plots, also called forest plots, were plotted with coefficient value, ln(odds ratio), with 95% confidence intervals as whiskers. Bar charts were provided with error bars when multiple values were present, and these bars represented standard errors. Bar level represent the mean variable value.

## Supplemental information

Document S1. Figures S1–S5

Table S1. Clinical and demographic tables for all cohorts, related to Figure 1

Table S2. Plasma protein and RNA analysis for infection cohorts, related to Figure 2

Table S3. Flow cytometry percentages of memory-biased and short lived effector cell (SLEC)-like populations from healthy donors and patients with lupus, related to Figure 3

Table S4. Differentially expressed RNA between NKG2A^+^ and NKG2C^+^ tumor-infiltrating CD8^+^ T cells from the pan-cancer single-cell dataset, related to Figure 4

Document S2. Article plus supplemental information
