# Supplementary material for: Integrative systems biology reveals NKG2A-biased immune responses correlate with protection in infectious disease, autoimmune disease, and cancer
Source: Cell Rep. 2024 Feb 29;43(3):113872. doi: 10.1016/j.celrep.2024.113872 (PMC10995767; doi:10.1016/j.celrep.2024.113872)
Supplement: Document S1. Figures S1–S5 [file mmc1.pdf]

Cell Reports, Volume *43*

Supplemental information

Integrative systems biology reveals NKG2A-biased immune responses correlate with protection in infectious disease, autoimmune disease, and cancer

Daniel G. Chen, Jingyi Xie, Jongchan Choi, Rachel H. Ng, Rongyu Zhang, Sarah Li, Rick Edmark, Hong Zheng, Ben Solomon, Katie M. Campbell, Egmidio Medina, Antoni Ribas, Purvesh Khatri, Lewis L. Lanier, Philip J. Mease, Jason D. Goldman, Yapeng Su, and James R. Heath

Supplementary figures

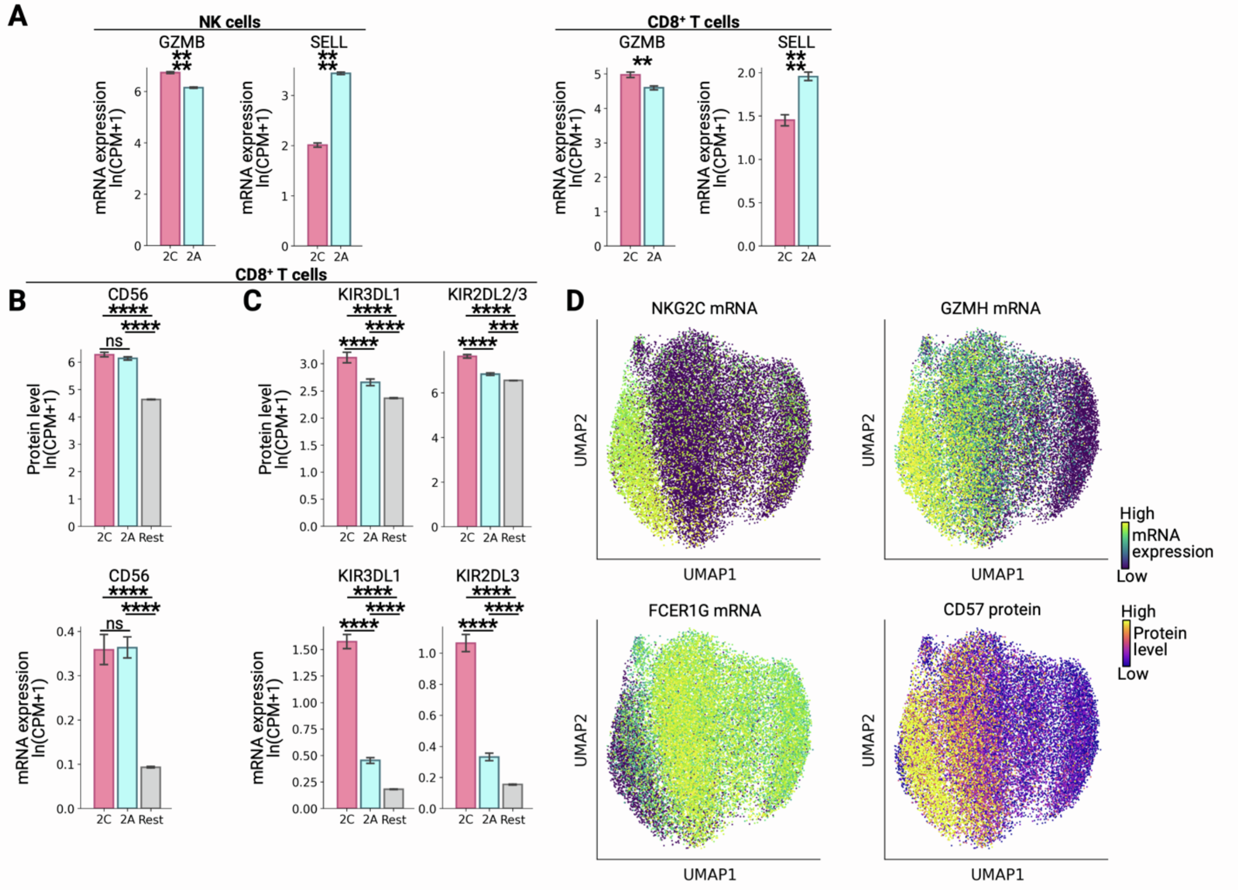

Supplementary Figure 1

Figure S1: NKG2A$^+$ and NKG2C$^+$ cells match previously described phenotypes

(a) Bar plots with x-axis as NKG2C$^+$ (2C, red) or NKG2A$^+$ (2A, blue) cells with y-axis as the normalized expression value of a given mRNA, labeled on the top of each bar plot. The cell type of origin for the cell subsets is labeled on the top of each pair of bar plots in bold and underline.

(b) (c) Bar plots with x-axis as the NKG2A/C subset and y-axis as the protein level, for the upper plot, or the mRNA expression, for the lower plot. Non-NKG2A/C$^+$ cells are labeled in grey as "rest", NKG2C$^+$ in red, and NKG2A$^+$ in blue.

(d) UMAP of single NK cells that are either NKG2A$^+$ or NKG2C$^+$ from COVID-19 patients and healthy controls. Select mRNA and proteins involved in defining adaptive NK cell phenotype are colored, see title and legend on the right.

Bar plots are presented as the mean value with standard error. P-values are annotated on all relevant plots with either value or stars, ****p<0.0001, ***p<0.001, **p<0.01, *p<0.05.

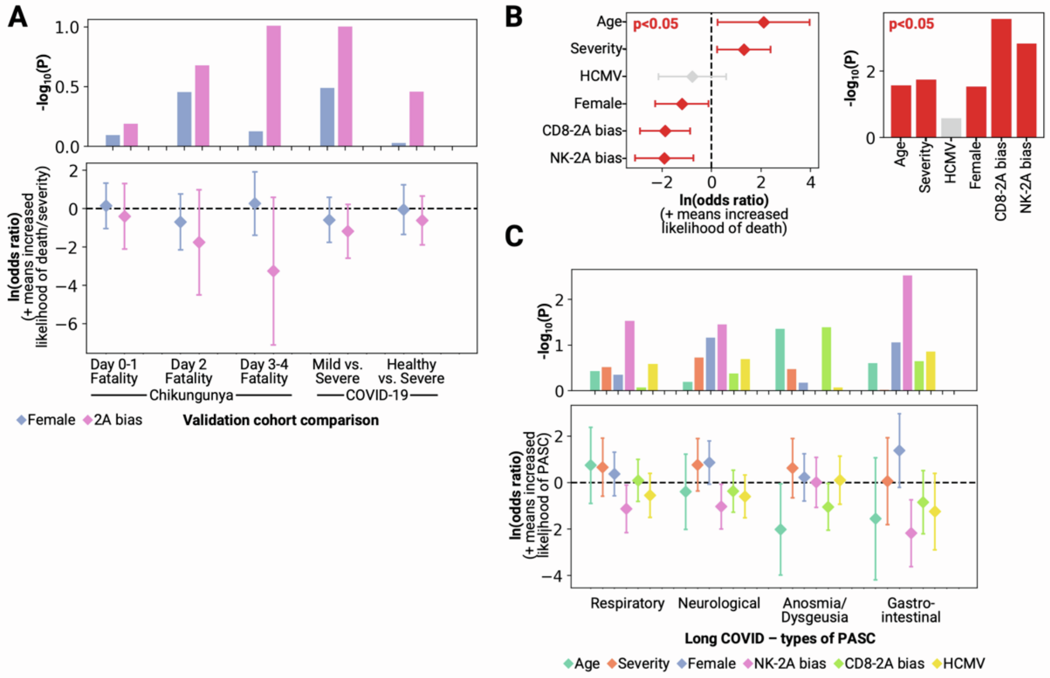

Supplementary Figure 2

Figure S2: External and internal validation of NKG2A$^+$ benefit across infectious disease contexts

(a) Log-odds model of mortality or severity, see lower labels, as predicted by NKG2A$^+$ bias while accounting for demographic factors. Upper: bar plot with the x-axis as different co-variates and symptoms, y-axis is the $-\log_{10}$(p-value). Lower: forest plot with the x-axis as different co-variates and symptoms, y-axis ln(odds ratio) for a given co-variate with 95% confidence intervals plotted as whiskers. Colors indicate different co-variates, see legend at bottom.

(b) Log-odds model of patient mortality predicted by NKG2A$^+$ bias while accounting for demographic factors. Left: forest plot with the y-axis as different co-variates, x-axis ln(odds ratio) for a given co-variate with 95% confidence intervals plotted as whiskers. Right: bar plot with the x-axis as different co-variates, y-axis is the $-\log_{10}$(p-value). Red color indicates significance, meaning $p < 0.05$.

(c) Log-odds model of whether a patient has a given long COVID symptom as predicted by NKG2A$^+$ bias while accounting for demographic factors. Upper: bar plot with the x-axis as different co-variates and symptoms, y-axis is the $-\log_{10}$(p-value). Lower: forest plot with the x-axis as different co-variates and symptoms, y-axis ln(odds ratio) for a given co-variate with 95% confidence intervals plotted as whiskers. Colors indicate different co-variates, see legend at bottom.

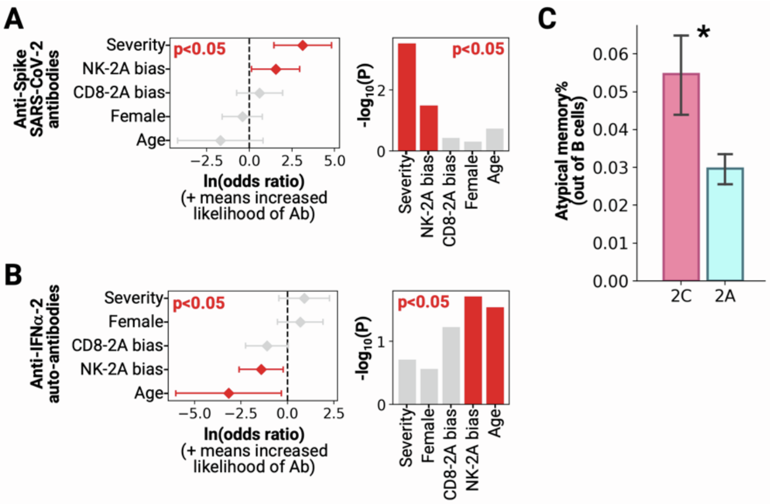

Supplementary Figure 3

Figure S3: NKG2A$^+$ bias associates positively with humoral immunity and negatively with auto-immunity related biomarkers

(a) Log-odds model of a given patient's titer of anti-spike SARS-CoV-2 antibodies 2-3 months after initial infection as predicted by NKG2A$^+$ bias while accounting for demographic factors. Left: forest plot with the y-axis as different co-variates, x-axis ln(odds ratio) for a given co-variate with 95% confidence intervals plotted as whiskers. Right: bar plot with the x-axis as different co-variates, y-axis is the -$\log_{10}$(p-value). Red color indicates significance, meaning $p < 0.05$.

(b) Log-odds model of a given patient's titer of anti-IFNα-2 auto-antibodies 2-3 months after initial infection as predicted by NKG2A$^+$ bias while accounting for demographic factors. Left: forest plot with the y-axis as different co-variates, x-axis ln(odds ratio) for a given co-variate with 95% confidence intervals plotted as whiskers. Right: bar plot with the x-axis as different co-variates, y-axis is the -$\log_{10}$(p-value). Red color indicates significance, meaning $p < 0.05$.

(c) Bar plot with x-axis as NKG2A$^+$, in blue, or NKG2C$^+$, in red, cells with y-axis as the percentage of a patient's B cells that bear an atypical memory phenotype.

Bar plots are presented as the mean value with standard error. P-values are annotated on all relevant plots with either value or stars, ****p<0.0001, ***p<0.001, **p<0.01, *p<0.05.

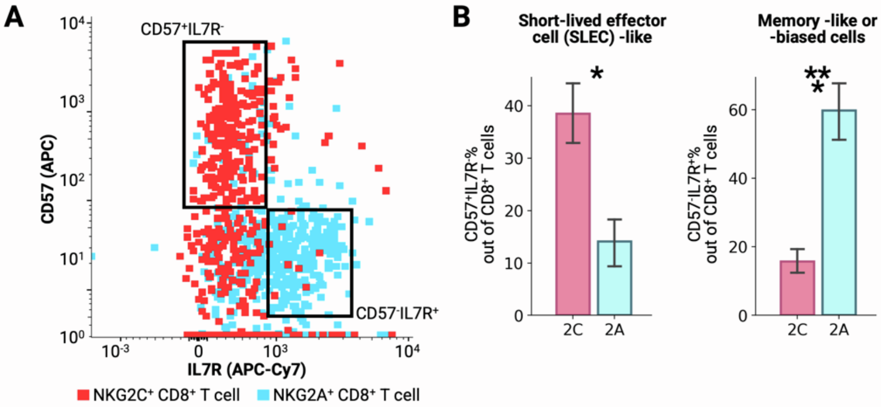

Supplementary Figure 4

Figure S4: Flow cytometry validation of phenotypic features of NKG2A/C subsets

a) Representative scatter plot of flow cytometry run from a given patient or donor with the x-axis as IL7R protein and y-axis as CD57 carbohydrate. Red dots are $NKG2C^+$ $CD8^+$ T cells, and blue dots are $NKG2A^+$ $CD8^+$ T cells. Each dot is a cell.
b) Bar plots for a given phenotype, derived from the black bounding boxes in panel A, out of a given patients $NKG2A/C^+$ $CD8^+$ T cells. For example, the red bar plot represents the percentage of $NKG2C^+$ $CD8^+$ T cells with that given phenotype.

Bar plots are presented as the mean value with standard error. P-values are annotated on all relevant plots with either value or stars, ****p<0.0001, ***p<0.001, **p<0.01, *p<0.05.

A

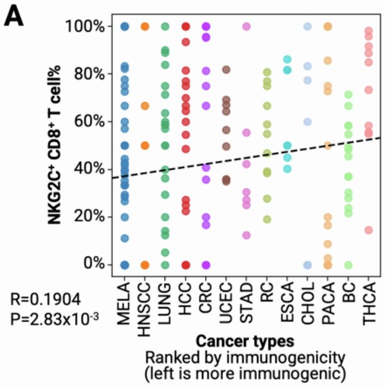

B

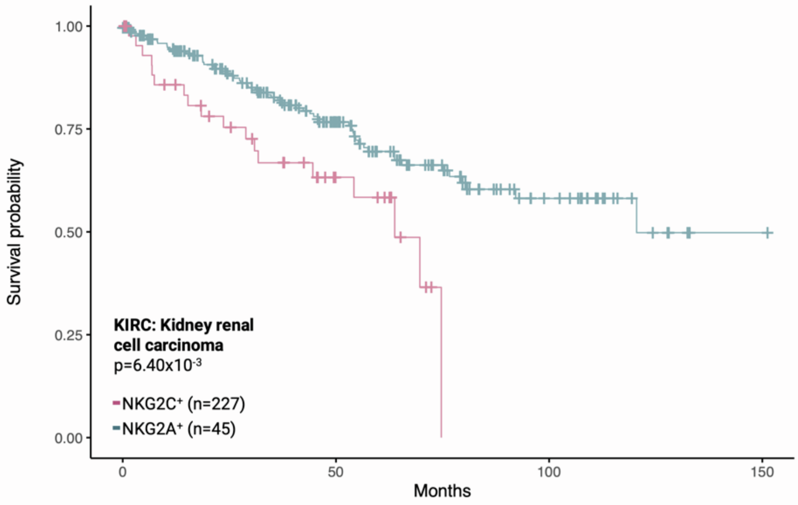

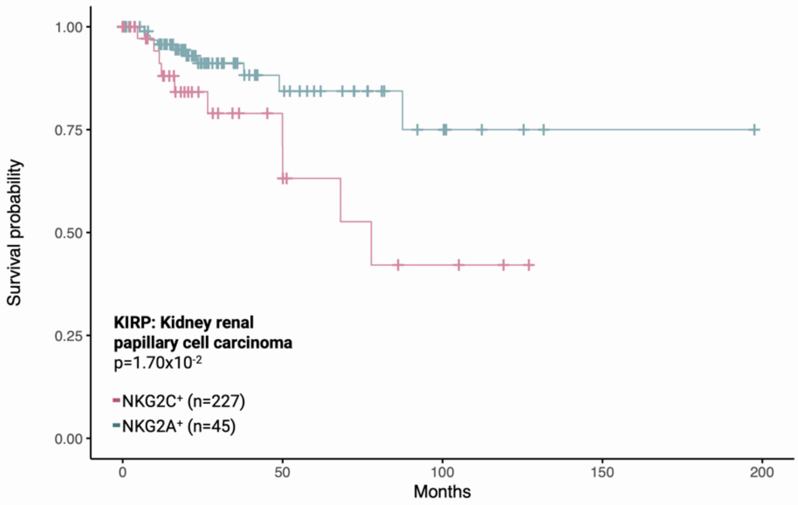

Supplementary Figure 5

Figure S5: Patients with an NKG2A$^+$ bias derive clinical benefit in cancer contexts

a) Scatter plot with the x-axis as different cancer types ranked by their tumor immunogenicity with increasing immunogenicity from left to right. The y-axis represents the percentage of a patient's tumor-infiltrating CD8$^+$ T cells that are NKG2C$^+$. Each dot is a patient. Fitted linear line is plotted in black dashes.
b) Kaplan-meier survival plots of NKG2A$^+$, in blue, versus NKG2C$^+$, in red, biased patients based on bulkRNA-seq data from TCGA patients. Log-rank test is used to calculate the displayed p-value. Each plot is for a single cancer type.

P-values are annotated on all relevant plots with value.

Supplementary information tables

Table S1 (related to Figure 1): clinical and demographic tables for all cohorts

Table S2 (related to Figure 2): plasma protein and RNA analysis for infection cohorts

Table S3 (related to Figure 3): flow cytometry percentages of memory biased and short lived effector cells (SLEC) -like populations from healthy donors and patients with lupus

Table S4 (related to Figure 4): differentially expressed RNA between $NKG2A^+$ and $NKG2C^+$ tumor-infiltrating $CD8^+$ T cells from the pan-cancer single cell dataset
